# Reduced CXCL4/PF4 expression as a driver of increased human hematopoietic stem and progenitor cell proliferation in polycythemia vera

**DOI:** 10.1038/s41408-021-00423-5

**Published:** 2021-02-11

**Authors:** Fabienne Meier-Abt, Witold E. Wolski, Ge Tan, Sandra Kummer, Sabine Amon, Markus G. Manz, Ruedi Aebersold, Alexandre P. A. Theocharides

**Affiliations:** 1 Department of Medical Oncology and Hematology, University Hospital Zurich and University of Zurich, Zurich, Switzerland; 2 Department of Biology, Institute of Molecular Systems Biology, ETH Zurich, Zurich, Switzerland; 3 Functional Genomics Center Zurich, University and ETH Zurich, Zurich, Switzerland; 4 Swiss Institute of Bioinformatics, Lausanne, Switzerland; 5 Faculty of Science, University of Zurich, Zurich, Switzerland

**Keywords:** Myeloproliferative disease, Preclinical research

Dear Editor,

Polycythemia vera (PV) is a myeloproliferative neoplasm (MPN) marked by hyperproliferation of all myeloid cell lineages and characterized by activated JAK-STAT signaling due to an activating mutation in *JAK2*^1^. Disease-driving pathogenic changes in MPNs are thought to arise in hematopoietic stem cells (HSCs) that give rise to the diseased clonal progeny^2,3^. We recently developed a new data-independent acquisition (DIA) mass spectrometry (MS) technology for rare human hematopoietic stem and progenitor cell (HSPC) subpopulations^4^. This DIA-MS proteomic analysis was applied to human hematopoietic stem/multipotent progenitor cells (HSC/MPPs) and common myeloid/megakaryocyte-erythrocyte progenitors (CMP/MEPs) isolated from 123 blood samples of 18 PV patients and 21 controls (Supplementary Table 1). The proteomic dataset was complemented with RNA-sequencing data of the same patient and control samples.

Comparing the proteomic and transcriptomic datasets in the corresponding patient and control samples demonstrated mainly positive correlations: 70% of genes had positive Spearman’s correlation coefficients for protein and RNA expression. In line with our previous observations in HSC/MPPs of healthy stem cell donors^4^, 30% of genes with altered expression in PV patients and their controls demonstrated negative correlation coefficients for protein and RNA expression, indicating additional information gained by proteomic compared to transcriptomic data.

## Downregulation of megakaryocyte differentiation and upregulation of cell proliferation in PV HSPCs and its reversal upon treatment with hydroxyurea (HU)

**Fig. 1:**
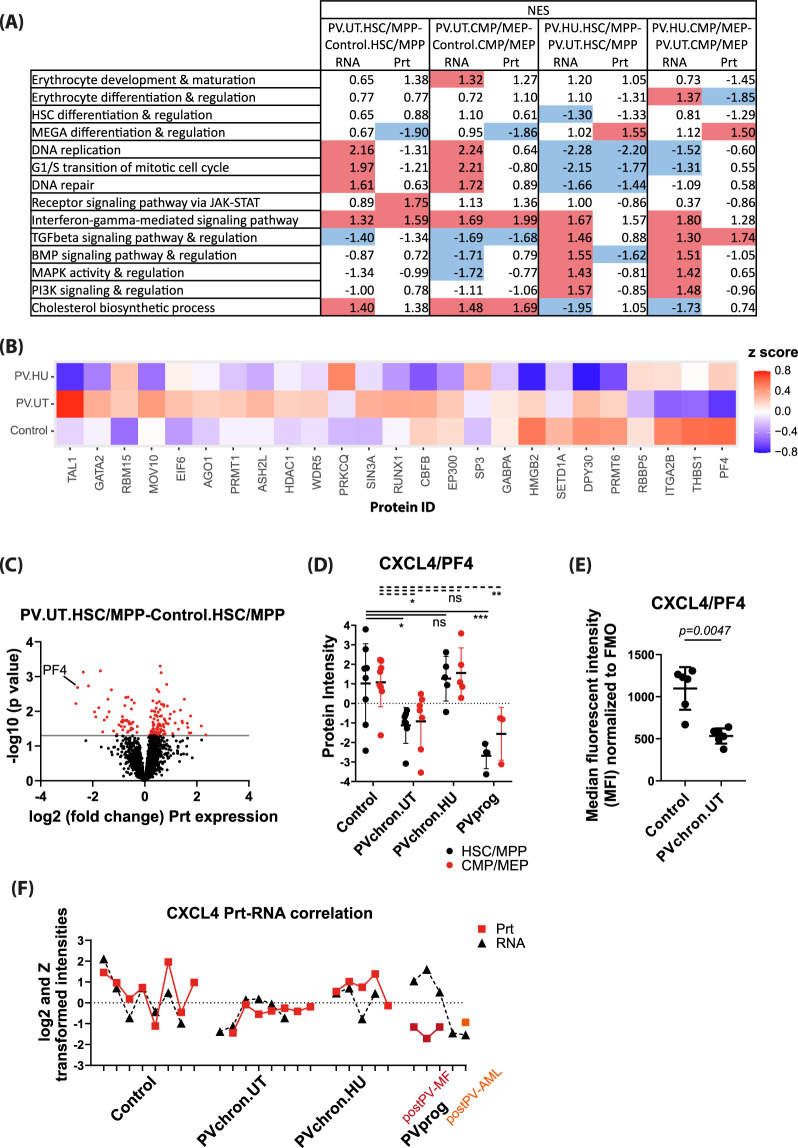
Comparative unbiased proteomic analysis identifies downregulation of CXCL4/PF4 in HSC/MPPs of PV patients. **A** Gene set enrichment analysis (GSEA) comparing untreated PV patients against controls (PV.UT.HSC/MPP versus Control.HSC/MPP; PV.UT.CMP/MEP versus Control.CMP/MEP) and assessing for the effect of treatment with hydroxyurea (PV.HU.HSC/MPP versus PV.UT.HSC/MPP; PV.HU.CMP/MEP versus PV.UT.CMP/MEP). Shown are normalized enrichment scores (NES) for individual gene sets. Significantly upregulated gene sets are marked in red color, significantly downregulated gene sets are marked in blue color. Only RNAs and proteins expressed in at least half of the replicates in both comparison groups were considered. UT untreated, HU patient under treatment with hydroxyurea. **B** Heatmap of proteins enriched in different patient and control groups for megakaryocyte differentiation and regulation. **C** Volcano plot of protein intensity fold changes and p-values comparing HSC/MPPs of untreated PV patients (PV.UT.HSC/MPP) against controls (Control.HSC/MPP). **D** Normalized protein intensities for CXCL4/PF4 in the subgroups of controls, chronic PV patients without cytoreductive therapy (PVchron.UT), chronic PV patients with hydroxyurea therapy (PVchron.HU), and progressed PV patients (PVprog). Error bars represent standard deviations. *adj. *P* < 0.05; **adj. *P* < 0.01; ***adj. *P* < 0.001 Peptide profiles for CXCL4/PF4 are provided in Supplementary Fig. 1. **E** Graphical summary of intracellular FACS staining experiments for CXCL4/PF4 in six untreated chronic PV patients and age- and gender-matched controls. Error bars represent standard deviations. **F** Normalized CXCL4/PF4 expression values for protein and RNA in individual patient and control samples. Discrepant high RNA and low protein levels were seen in progressed PV patients with fibrosis (post-PV MF) but not in post-PV AML.

To further examine the added information provided by protein compared to RNA expression data, we performed enrichment analyses for gene ontologies in HSPC subpopulations of PV patients on protein and RNA levels. Megakaryocyte differentiation and regulation was significantly downregulated in untreated PV patients at the protein level, but not at the RNA level in all HSPCs analyzed (Fig. 1A with individual gene ontology protein members shown in Fig. 1B). Similarly, RNAs and proteins implicated in DNA replication and G1/S transition of the mitotic cell cycle were discordantly regulated in HSC/MPPs of untreated PV patients (Fig. 1A). In contrast to the discordant protein and RNA expression in these gene sets, we observed concordant expression on the protein and RNA level for erythrocyte differentiation, regulation, development and maturation, receptor signaling via JAK-STAT, interferon-gamma signaling, cholesterol biosynthetic process, and TGFβ and MAPK signaling and regulation in HSPCs of untreated PV patients compared to controls (Fig. 1A).

Cytoreductive therapy with hydroxyurea reverted downregulation of megakaryocyte differentiation and regulation on protein level (Fig. 1A, B). It also reversed upregulation of cell proliferation (DNA replication, G1/S transition of the mitotic cell cycle, DNA repair) on RNA level and downregulation of TGFβ signaling on protein and RNA level (Fig. 1A). Erythrocyte differentiation and regulation were reversed by hydroxyurea on the protein but not RNA level, whereas interferon-gamma signaling was not affected by the treatment of patients with hydroxyurea (Fig. 1A).

### Reduced CXCL4/PF4 expression in HSC/MPPs of untreated PV patients

To better characterize the molecular phenotype underlying PV stem and progenitor cell biology, we next focused on individual differentially regulated proteins in HSC/MPPs of untreated PV patients compared to controls. Consistent with the downregulation of megakaryocyte differentiation and regulation observed in the gene ontology enrichment analysis (Fig. 1A, B), we observed significant downregulation of the megakaryocytic lineage protein CXCL4/PF4 (platelet factor 4) (Fig. 1C, D and Supplementary Fig. 1). Intracellular flow cytometry confirmed the downregulation of CXCL4/PF4 in HSC/MPPs of untreated chronic phase PV patients in comparison to age- and gender-matched controls (Fig. 1E). Treatment with hydroxyurea abrogated the downregulation of CXCL4/PF4 protein expression in PV HSC/MPPs (Fig. 1D).

Since all PV patients analyzed in this study carried the *JAK2V617F* mutation, we tested for a potential direct relationship between the *JAK2V617F* allele burden and CXCL4/PF4 protein expression. No significant correlation was observed (Supplementary Fig. 2), suggesting that reduced CXCL4/PF4 expression in PV is a *JAK2V617F* allele burden-independent disease manifestation.

CXCL4/PF4 did not show equally significant expression changes on the RNA level as on the protein level. Exploring protein-RNA-correlations in more detail, we observed that protein and RNA levels became discordant upon disease progression (Fig. 1F). Upregulated RNA and downregulated protein levels for CXCL4/PF4 were observed in HSC/MPPs of post-PV myelofibrosis (MF) patients but not in post-PV acute myeloid leukemia (AML) stem cells. In controls and non-progressed PV patients, protein and RNA levels of CXCL4/PF4 were better aligned even if not systematically coordinated (Fig. 1F).

### Decreased ELF1 and USF2 activity explains reduced CXCL4/PF4 expression, which is linked to downregulated TGFβ signaling and loss of stem cell quiescence

**Fig. 2:**
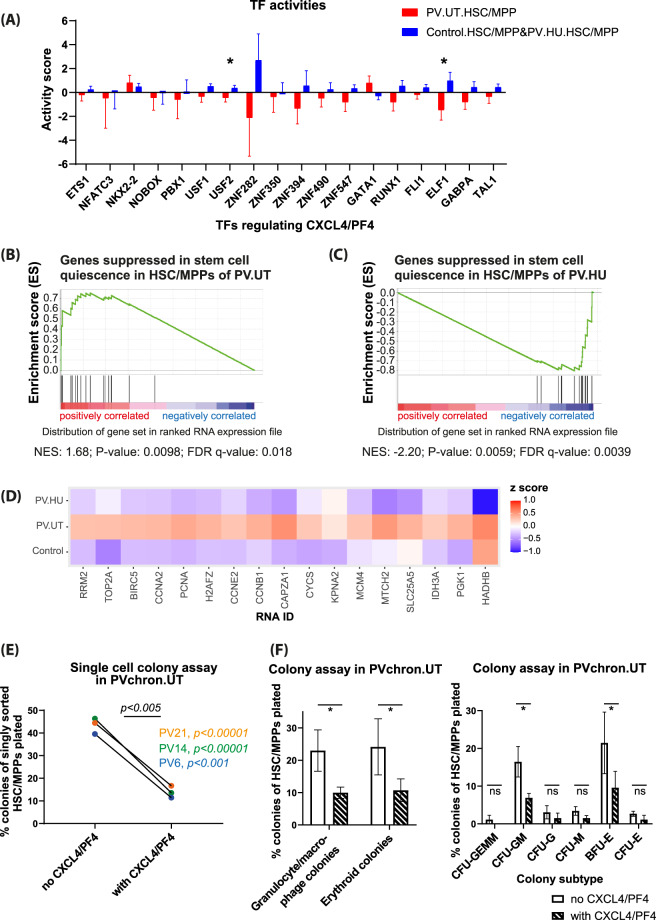
CXCL4/PF4 protein is associated with transcriptional stem cell quiescence in untreated PV and inhibits the colony-formation capacity of PV HSC/MPPs. **A** Activity analysis of transcription factors (TFs) regulating CXCL4/PF4 expression showed significantly downregulated activity for the TF ELF1 and to a lesser degree for the TF USF2 in untreated PV patients compared to controls and HU-treated PV patients. Error bars represent standard errors. *P < 0.05. **B** Gene set enrichment analysis showed strong and significant enrichment for genes downregulated in stem cell quiescence in HSC/MPPs of untreated PV patients (PV.UT) compared to HSC/MPPs of controls, suggesting cessation of stem cell quiescence in PV stem cells. **C** Treatment of patients with hydroxyurea (PV.HU.HSC/MPP versus PV.UT.HSC/MPP) reversed the loss of stem cell quiescence in the PV HSC/MPP subpopulation. NES normalized enrichment score. **D** Heatmap of RNAs enriched in different patient/control groups for genes downregulated in stem cell quiescence. The corresponding enrichment plots and heatmap for the protein level are shown in Supplementary Fig. 4A-C. **E** Functional single-cell CXCL4/PF4 assay: colony growth of FACS-sorted HSC/MPPs from untreated chronic PV patients (PVchron.UT) singly incubated in cytokine-enriched serum-free medium was assessed in presence or absence of CXCL4/PF4. Colony growth as depicted was evaluated in 864 wells after 7 days of incubation. In individual patients, significance was determined using Fisher’s exact test; significance overall was calculated using two-tailed and paired Student’s *t* test (see Supplementary Methods). A strong growth inhibitory role of CXCL4/PF4 was observed. **F** Methylcellulose colony assay: the growth of different myeloid colony subtypes from FACS-sorted HSC/MPPs of untreated chronic PV patients (PVchron.UT) was evaluated in presence or absence of CXCL4/PF4. Reductions in colony growth due to CXCL4/PF4 protein were observed for all colony subtypes with significant inhibitions seen in the CFU-GM and BFU-E colony subtypes as well as in composite granulocyte/macrophage (CFU-GM and CFU-G and CFU-M) and erythroid (BFU-E and CFU-E) colonies. Relative numbers of colonies related to the total cell numbers plated are plotted. Error bars represent standard deviations. *P < 0.05. CFU-GEMM (mixed colonies), CFU-GM colony-forming unit–granulocyte/macrophage, CFU-G colony-forming unit–granulocyte, CFU-M colony-forming unit–macrophage, BFU-E burst-forming unit–erythroid, CFU-E colony-forming unit–erythroid.

We next assessed the activities of transcription factors (TFs), which bind the CXCL4/PF4 promoter and may regulate CXCL4/PF4 expression in PV HSC/MPPs (Fig. 2A)^5–8^. We found a significantly decreased activity for ELF1 and to a lesser extent also for USF2 in HSC/MPPs of untreated PV compared to controls and HU-treated PV (Fig. 2A), suggesting a potential link between diminished ELF1 and USF2 activity and a reduction of CXCL4/PF4 expression in HSC/MPPs of untreated PV patients^5,8^. Upon progression to post-PV MF, the activities of the TFs USF2 and also NKX2-2 increased, potentially explaining upregulated RNA levels of CXCL4/PF4 in these patients (Supplementary Fig. 3)^5^.

Reduced CXCL4/PF4 expression in HSC/MPPs of PV patients was associated with significant upregulation of genes downregulated in stem cell quiescence both on RNA (Fig. 2B, D) and protein (Supplementary Fig. 4A and 4C) level^9^. This pattern was reversed by treatment of patients with cytoreductive hydroxyurea (Fig. 2C, D and Supplementary Fig. 4B, C). Mechanistically, CXCL4/PF4 upregulates TGFβ^10^, which promotes stem cell quiescence^11^. We observed downregulation of TGFβ signaling in untreated PV patients that also exhibited downregulated CXCL4/PF4 levels (Fig. 1A). These data suggest that reduced CXCL4/PF4 expression in PV HSC/MPPs leads to the cessation of stem cell quiescence via downregulation of TGFβ signaling.

### CXCL4/PF4 inhibits colony formation of HSC/MPPs isolated from untreated chronic phase PV patients

To assess the functional relevance of CXCL4/PF4 downregulation and to examine its causal link to HSC/MPP proliferation and differentiation in PV, we cultured HSC/MPPs of untreated chronic phase PV patients in the presence or absence of CXCL4/PF4 using two independent colony formation assays. Supplementation with CXCL4/PF4 inhibited colony formation of singly sorted PV HSC/MPPs after 7 days in the cytokine-enriched serum-free medium by 63–71% (Fig. 2E). In methylcellulose assays, a 54–61% inhibition of total colony formation was observed upon reconstitution/treatment with CXCL4/PF4 for FACS-sorted HSC/MPPs from PV patients (Fig. 2F). Detailed analyses for colony subtypes as defined by Manz et al.^12^ showed significant reductions in colony growth from PV HSC/MPPs upon exposure to supplemental CXCL4/PF4 for CFU-GM (colony-forming unit–granulocyte/macrophage) and BFU-E (burst-forming unit–erythroid) (Fig. 2F). These results demonstrate that CXCL4/PF4 is linked to HSC/MPP proliferation and differentiation in PV, and reduced CXCL4/PF4 expression in HSC/MPPs may contribute to the proliferative state of PV.

## Discussion

This study identifies reduced CXCL4/PF4 protein expression in HSC/MPPs of untreated PV patients compared to controls. Treatment of patients with cytoreductive hydroxyurea abrogated this effect. We demonstrated transcriptional cessation of stem cell quiescence to be associated with reduced CXCL4/PF4 expression in PV HSC/MPPs, and supplementation with CXCL4/PF4 strongly inhibited the in vitro colony-formation capacity of HSC/MPPs from PV patients. These findings extend previous reports in healthy hematopoietic stem cells and non-PV *JAK2*-mutated erythroblast-like cells^13^. CXCL4/PF4-/- mice were shown to exhibit increased numbers and proliferation of HSCs and MPPs^14^, and high CXCL4/PF4 levels were found to strongly inhibit hematopoiesis in non-PV mice^14^. The inhibitory role of CXCL4/PF4 on HSPC proliferation in our PV patients supports a key role of downregulated CXCL4/PF4 for the proliferative phenotype of PV HSC/MPPs.

Mechanistically, we observed downregulation of TGFβ signaling in untreated PV patients showing reduced CXCL4/PF4 expression. CXCL4/PF4 was previously shown to activate stem cell quiescence-inducing TGFβ^10,11^. Upstream of CXCL4/PF4, we identified reduced activity of the CXCL4/PF4-regulating transcription factors ELF1^8^ and USF2^5^ in HSC/MPPs of untreated PV patients. Our data thus support a model in which decreased ELF1 and USF2 activity in PV HSC/MPPs leads to reduced CXCL4/PF4 expression, which leads to decreased TGFβ signaling and concomitant cessation of stem cell quiescence. Upon progression to post-PV MF, USF2 activity increased, explaining elevated CXCL4/PF4 RNA levels in this patient group.

The PV disease stages and controls were better separated by CXCL4/PF4 protein expression than by CXCL4/PF4 RNA expression. Whereas transcriptomics reinforced the proteomic results for CXCL4/PF4 in chronic phase PV, discrepant RNA and protein levels were observed in HSC/MPPs of post-PV MF patients. Our findings in fibrotic patients are in line with recent observations of upregulated CXCL4/PF4 RNA levels in co-cultured HSPCs of a murine PMF model^15^. The congruent CXCL4/PF4 protein and RNA expression in control, chronic PV, and post-PV AML, but not in post-PV MF suggests post-transcriptional regulation at the base of discrepant RNA and protein levels in post-PV MF.

In summary, this study identified downregulation of CXCL4/PF4 expression in HSC/MPPs with the cessation of stem cell quiescence through downregulation of TGFβ signaling as a potential new driver of the proliferative state of PV.

## Supplementary information

Supplemental material

## Data Availability

Proteomic and transcriptomic data can be made available upon request to the corresponding author.
