# Supplementary material for: Reduced CXCL4/PF4 expression as a driver of increased human hematopoietic stem and progenitor cell proliferation in polycythemia vera
Source: Blood Cancer J. 2021 Feb 11;11(2):31. doi: 10.1038/s41408-021-00423-5 (PMC7878875; doi:10.1038/s41408-021-00423-5)
Supplement: Supplementary file 1 — Supplemental material [file 41408_2021_423_MOESM1_ESM.pdf]

Supplementary Table 1						
**PROTEOMIC/TRANSCRIPTOMIC ANALYSES**						
**Group**	**Subgroups**	**Patient**	**Age**	**Sex**	**Diagnosis**	**Therapy**
PV	PVchron.UT	PV1	57	m	PV	ASA, BP
PV	PVchron.UT	PV5	59	m	PV	ASA
PV	PVchron.UT	PV6	53	m	PV	ASA
PV	PVchron.UT	PV8	68	f	PV	ASA, BP, statin
PV	PVchron.UT	PV9	55	m	PV	ASA, BP
PV	PVchron.UT	PV12	44	m	PV	ASA, BP
PV	PVchron.UT	PV13	55	m	PV	ASA, BP
PV	PVchron.UT	PV14	44	f	PV	ASA, anti-dep
PV	PVchron.UT	PV16	50	m	PV	ASA
PV	PVchron.HU	PV2	65	m	PV	HU, ASA, BP, statin
PV	PVchron.HU	PV3	77	m	PV	HU, ASA, BP
PV	PVchron.HU	PV4	57	m	PV	HU, ASA, BP
PV	PVchron.HU	PV5	60	m	PV	HU, ASA
PV	PVchron.HU	PV7	79	m	PV	HU, ASA, BP, anti-epi
PV	PVprog.HU	PV10	65	m	PV transforming into AML	HU, ASA
PV	PVprog.HU	PV11	61	m	Post-PV MF	HU, ASA
PV	PVprog.HU	PV15	82	m	Post-PV MF	HU, phenprocoumon, anti-uric
PV	PVprog.UT	PV17	64	m	Post-PV MF	BP, anti-uric, pred
PV	PVprog.UT	PV18	72	f	PV transforming into MF	ASA, BP, anti-uric
Control	Control	CON1	45-74	m,f	4 Healthy Blood Donors	n.a.
Control	Control	CON2	45-74	m,f	4 Healthy Blood Donors	n.a.
Control	Control	CON3	45-74	m,f	4 Healthy Blood Donors	n.a.
Control	Control	CON4	71	m	Hemochromatosis	BP
Control	Control	CON5	42	m	Hemochromatosis	none
Control	Control	CON5	66	m	Hemochromatosis	ASA, BP, anti-dep
Control	Control	CON5	61	f	Hemochromatosis	inhal
Control	Control	CON5	40	m	Hemochromatosis	none
Control	Control	CON6	54	m	Hemochromatosis	ASA, anti-diab
Control	Control	CON7	45	m	Hemochromatosis	thyrox
Control	Control	CON7	60	f	Hemochromatosis	BP, HRT
Control	Control	CON8	63	m	Hemochromatosis	statin
**VALIDATION EXPERIMENTS**						
**Group**	**Subgroups**	**Patient**	**Age**	**Sex**	**Diagnosis**	**Therapy**
PV	PVchron.UT	PV6	47	m	PV	ASA
PV	PVchron.UT	PV9	53	m	PV	ASA, BP
PV	PVchron.UT	PV13	55	m	PV	ASA
PV	PVchron.UT	PV14	43	f	PV	ASA, Infliximab
PV	PVchron.UT	PV19	49	f	PV	Clopidogrel
PV	PVchron.UT	PV21	68	m	PV	ASA
PV	PVchron.UT	PV22	44	m	PV	ASA, BP
PV	PVchron.UT	PV23	55	m	PV	ASA
PV	PVchron.UT	PV24	33	m	PV	ASA, anti-dep
PV	PVprog.RU	PV11	62	m	Post-PV MF	Ruxolitinib, ASA
PV	PVprog.UT	PV18	71	f	PV transforming into MF	ASA, BP
PV	PVprog.UT	PV20	77	m	Post-PV AML	ASA, BP, anti-uric

Control	Control	CON9	54	m	Healthy Blood Donor	n.a.
Control	Control	CON10	55	m	Healthy Blood Donor	n.a.
Control	Control	CON11	56	m	Healthy Blood Donor	n.a.
Control	Control	CON12	74	m	Healthy Blood Donor	n.a.
Control	Control	CON13	45	f	Healthy Blood Donor	n.a.
Control	Control	CON14	53	f	Healthy Blood Donor	n.a.
Control	Control	CON15	59	m	Healthy Blood Donor	n.a.
Control	Control	CON16	46	f	Healthy Blood Donor	n.a.
Control	Control	CON17	57	m	Healthy Blood Donor	n.a.
Control	Control	CON18	53	m	Healthy Blood Donor	n.a.
Control	Control	CON19	62	m	Healthy Blood Donor	n.a.
Control	Control	CON20	65	f	Healthy Blood Donor	n.a.
Control	Control	CON21	45-74	m,f	Healthy Blood Donor	n.a.
Control	Control	CON22	45-74	m,f	Healthy Blood Donor	n.a.
Control	Control	CON23	45-74	m,f	Healthy Blood Donor	n.a.

Abbreviations: PV – polycythemia vera, MF – myelofibrosis, AML – acute myeloid leukemia, chron – chronic, prog – progressed, UT – untreated, HU – under treatment with hydroxyurea, RU – under treatment with ruxolitinib, ASA – acetylsalicylic acid, HU – hydroxyurea, BP – blood pressure medication, anti-dep – anti-depressive medication, anti-epi – anti-epileptic medication, anti-uric – uric acid lowering medication, pred – prednison, inhal – inhalative medication, anti-diab – anti-diabetic medication, thyrox – thyroxine medication, HRT – hormone replacement therapy, m – male, f – female

Supplementary Table 1. Characteristics of PV patients and controls.

Supplementary Figure 1

CXCL4/PF4: Peptide profile as basis of CXCL4/PF4 protein quantification

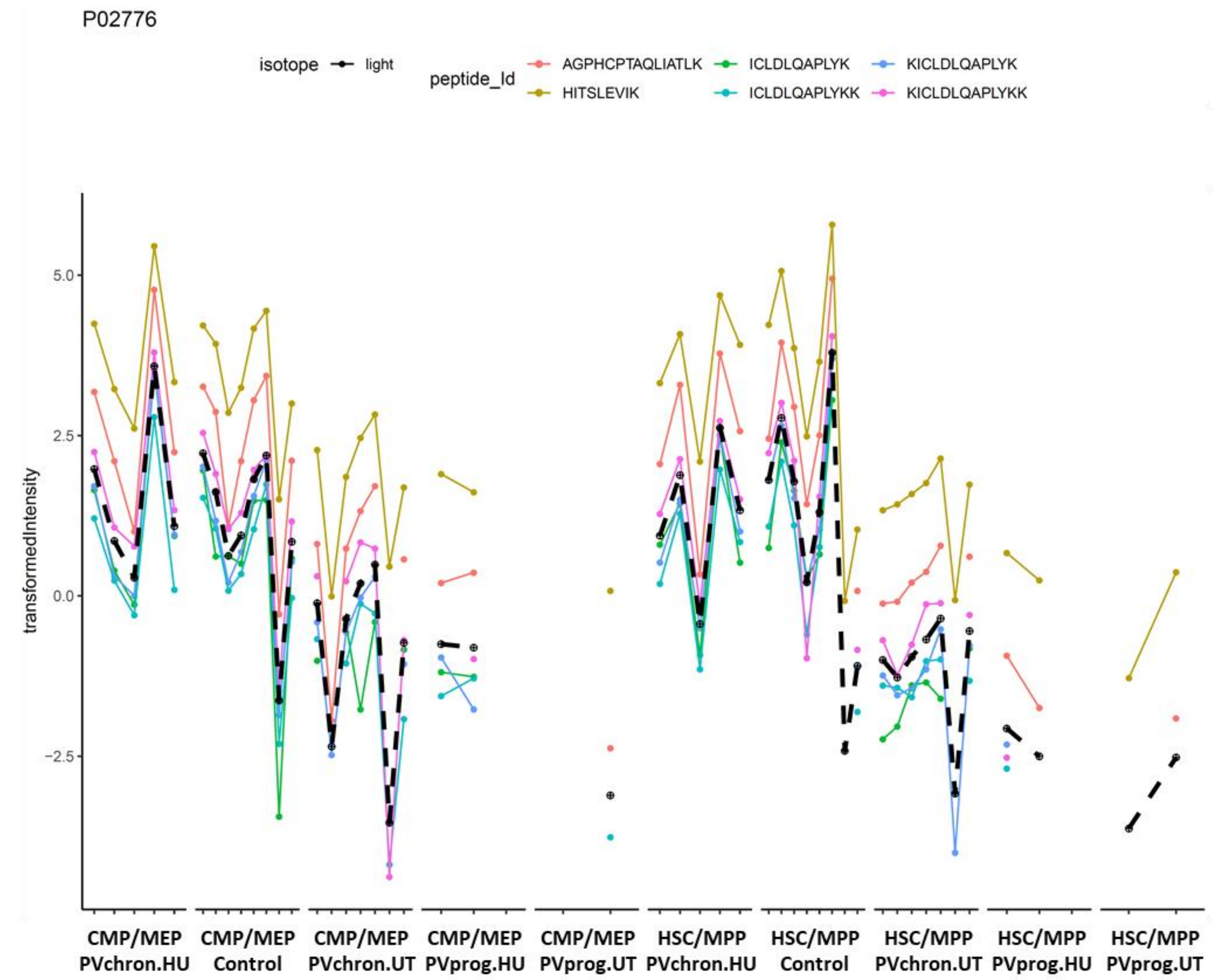

Supplementary Figure 2

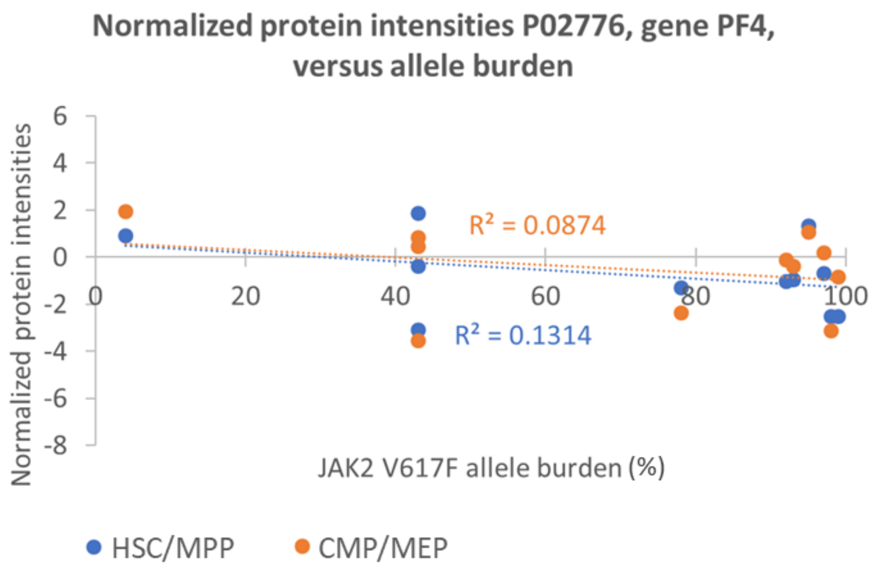

Supplementary Figure 2. Test of correlation of normalized protein intensities against *JAK2 V617F* allele burden for CXCL4/PF4. Normalized protein intensities were measured in HSC/MPPs and CMP/MEPs of PV patients. *JAK2 V617F* allele burden as depicted was determined by ddPCR in granulocytes and validated by RNA-seq in HSC/MPPs and CMP/MEPs of the same patients. No significant correlations were observed.

Supplementary Figure 3

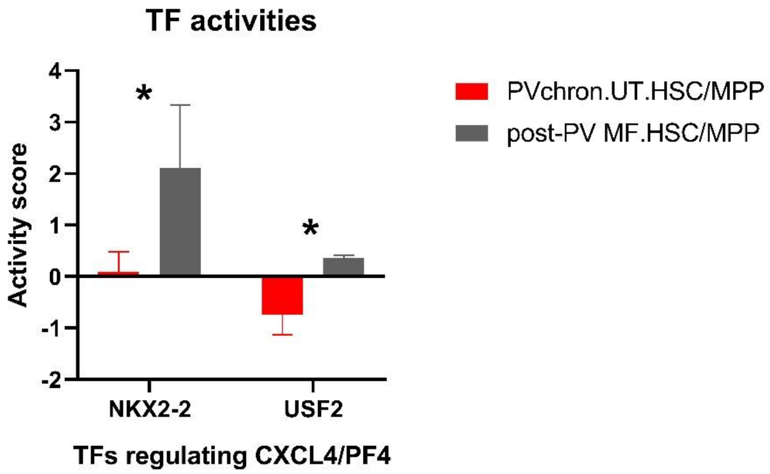

Supplementary Figure 3. Transcription factors (TFs) regulating CXCL4/PF4 expression with significant increases in activity in HSC/MPPs of post-PV MF compared to untreated chronic PV patients. Error bars represent standard errors. * $p < 0.05$

Supplementary Figure 4

(A) Proteins suppressed in stem cell quiescence in HSC/MPPs of PV.UT

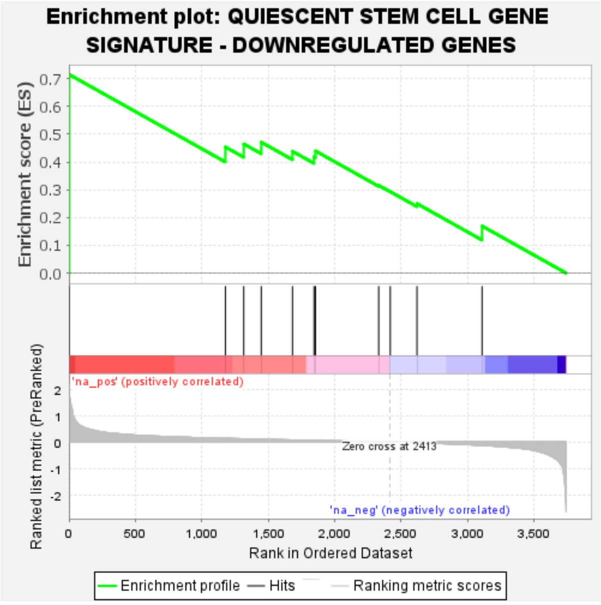

NES: 1.61
P-value: 0.0223
FDR q-value: 0.0554

(B) Proteins suppressed in stem cell quiescence in HSC/MPPs of PV.HU

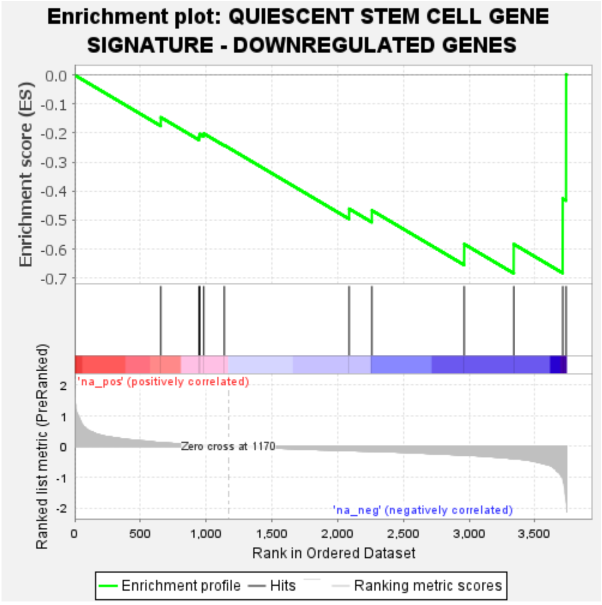

NES: -1.61
P-value: 0.0191
FDR q-value: 0.0149

(C)

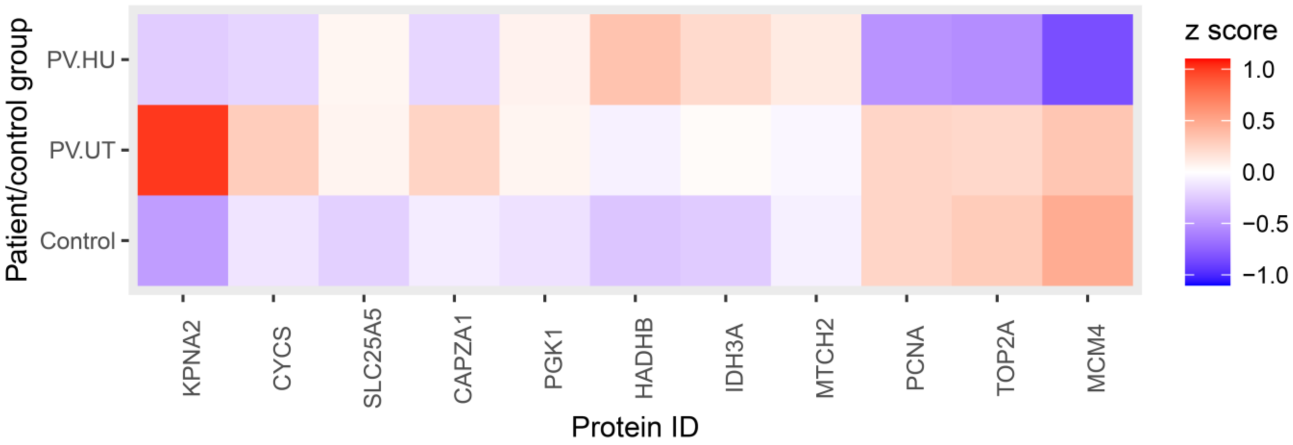

Supplementary Figure 4. Identification of loss of stem cell quiescence in HSC/MPPs of untreated PV patients compared to controls. (A) Gene set enrichment analysis showed significant enrichment for proteins downregulated in stem cell quiescence in HSC/MPPs of untreated PV patients (PV.UT) compared to HSC/MPPs of controls, thereby suggesting cessation of stem cell quiescence in PV stem cells. **(B)** Treatment of patients with hydroxyurea (PV.HU.HSC/MPP versus PV.UT.HSC/MPP) reversed loss of stem cell quiescence in the PV HSC/MPP subpopulation. Abbreviation: NES – normalized enrichment score. **(C)** Heatmap of proteins enriched in different patient and control groups for genes downregulated in stem cell quiescence.

METHODS

Human hematopoietic stem/progenitor cell samples. Fresh human peripheral blood samples were collected from patients with chronic and progressed PV (i.e. post-PV myelofibrosis (post-PV MF) or post-PV acute myeloid leukemia (post-PV AML)) and from controls with phlebotomy-requiring hemochromatosis during clinical routine phlebotomy appointments (Department of Medical Oncology and Hematology, University Hospital Zurich, Zurich, Switzerland). Buffy coat samples from healthy controls were obtained from the blood donation center SRK in Schlieren, Zurich. All samples were obtained with informed consent, and the studies were approved by the responsible local ethics committee (Kanton Zurich, Switzerland).

There were four subject groups: (i) chronic PV patients without cytoreductive treatment (PVchron.UT), (ii) chronic PV patients treated with hydroxyurea (HU) (PVchron.HU), (iii) progressed PV patients (PVprog.UT and PVprog.HU), and (iv) controls (hemochromatosis and buffy coats from healthy controls) (see supplementary table 1). Sample sizes for adequate statistical power were estimated on the basis of previous studies in healthy HSPCs (1).

Cell preparation, flow-cytometric analysis, cell sorting and sample preparation for mass spectrometry. Human hematopoietic stem cell-enriched subfractions and myeloid progenitors were isolated using fluorescence-activated cell sorting (FACS) as previously described (1). For flow-cytometric analysis of intracellular CXCL4/PF4 expression, the IntraPrep Leucocytic Permeabilization Reagent Kit (Beckman Coulter, Nyon, Switzerland) was used together with PE-conjugated anti-CXCL4/PF4, 170138 (R&D Systems, Zug, Switzerland) and a PE-conjugated isotype control. Dead cells were excluded in the analysis by staining with Zombie Aqua Fixable Viability Stain (BioLegend, Koblenz, Germany). A complete list of antibodies used is provided below.

For mass spectrometry (MS) analysis, 25,000 HSC/MPPs and CMP/MEPs were sorted into protein low-binding micro-centrifuge tubes (Eppendorf, Schönenbuch, Switzerland) and processed for MS as previously described (1). For RNA-seq analysis, up to 10,000 HSC/MPPs, CMP/MEPs, CMPs, GMPs and MEPs were sorted into RNeasy lysis buffer (Qiagen, Hombrechtikon, Switzerland) with beta-

mercaptoethanol. In rare cases, phlebotomies from different individuals had to be pooled in order to ensure adequate HSC/MPP numbers for downstream MS and RNA-seq measurements (see supplementary table 1).

Mass spectrometry analysis. Two MS analysis methods were employed. For generation of the spectral library required for subsequent analyses of proteins from FACS-isolated clinical cell samples, a data-dependent acquisition (DDA) mode of operation was used. For MS analysis of highly enriched human HSC/MPPs and CMP/MEPs from PV patients and controls, a data-independent acquisition (DIA) mode of operation was applied. These MS analysis methods were performed as recently described (1).

Proteomic data analysis. DDA data was searched by Mascot (2) (Matrix Science, version 2.5.1) as well as Comet (3) version 2016.01 rev. 2 against the Swissprot reviewed subset of the human UniProt database with decoy sequences generated by sequence reversal (keeping C-terminal K and R residues). Spectral libraries from DDA runs were generated as previously specified (4). DIA data was evaluated by Spectronaut 11 (5) (Biognosys, Schlieren, Switzerland), querying the library created from DDA runs.

Peptide intensities were log2 transformed and normalized with a modified robust z-score transformation. For comparative analysis, data was filtered for proteins with two or more proteotypic peptides, and a multilevel model was fitted to model differences among conditions per protein (6). The empirical Bayes approach implemented in the R package limma (7) was used to account for parallel measurements of several thousand proteins. To adjust for multiple testing and to estimate false discovery rates (FDR), the Benjamini Hochberg procedure was employed. No imputation was allowed for statistical analyses or graphs.

RNA isolation and sequencing. Total RNA was purified according to manufacturer's instructions using the RNeasy Plus Micro Kit (Qiagen). RNA sequencing was performed as described in Picelli et al (8) using the Illumina NovaSeq sequencing platform.

RNA-seq data analysis. Adapters and low-quality tails were trimmed from reads before mapping to transcriptome. STAR aligner (v2.6.1.c) (9) was used to align the RNA-seq data to Ensembl release 91 reference genome build GRCh38.p10. Gene expression values were quantified by featureCounts from the Bioconductor package Rsubread (v1.32.4) (10). Differential gene expression was analyzed with DESeq2 package (v1.22.2) (11).

Buffy coat samples were excluded from transcriptome analysis because they clustered away from other samples in RNA-seq but not in proteomic data, and pathway analysis of buffy coat versus other samples demonstrated RNA decay, but not protein decay, to be significantly upregulated in buffy coat samples.

Gene ontology enrichment analysis. For gene set enrichment analysis (GSEA), gene sets were retrieved from the Gene Ontology Consortium database (www.geneontology.org) on 2019-05-26 and from Cheung et al (12) (stem cell quiescence). Ranked lists were created from the normalized and filtered proteome and transcriptome data using log2 (fold change) as ranking criterion. GSEA was performed on the pre-ranked lists using the GSEA software (v3.0, http://www.broadinstitute.org/gsea) with default settings and minimal gene set size of 15 for transcriptomics and 5 for proteomics (in proportion to total identified targets). Enrichments were deemed significant when FDR < 0.25 as suggested by Subramanian et al (13).

Transcription factor activity analysis. The transcription factor (TF) and target interactions were obtained from DoRothEA (14), where the regulons were curated and inferred from various sources of evidence. The statistical tool viper (15) was used to predict TF activities.

Variant allele frequency (VAF) determination in granulocytes using droplet digital PCR (ddPCR). Granulocytes were isolated from PV patients using centrifugation followed by treatment with 150mM NH_4Cl/10mM $KHCO_3$/0.1mM NA_2EDTA to remove contaminating erythrocytes. Genomic DNA (gDNA) was extracted using the QIAamp DNA Mini Kit (Qiagen) according to manufacturer's instructions. ddPCR

for *JAK2-V617F* VAF determination was performed employing the BioRad QX200 ddPCR system, the assay dHsaMDV2010061 (BioRad, Basel, Switzerland) and the manufacturer's protocol.

Functional single cell CXCL4/PF4 assay. HSC/MPPs from three untreated chronic PV patients were singly sorted into 96-well plates. Wells were filled with 50uL of StemSpan SFEM (Stem Cell Technologies, Köln, Germany) supplemented with 100ng/mL SCF (Thermofischer Scientific, Basel, Switzerland), 100ng/mL FLT3L (Thermofischer Scientific), 100ng/mL TPO (PeproTech, London, United Kingdom), 20ng/mL IL6 (Thermofischer Scientific) and 50ng/mL IL3 (Thermofischer Scientific), as well as with or without 50ug/mL CXCL4/PF4 (Peprotech) (16). Cells to be sorted into wells with CXCL4/PF4 were pre-incubated with CXCL4/PF4 (50ug/mL) in FACS buffer for approx. 30min prior to sorting into cytokine containing medium. A total of 864 wells were evaluated for colony growth after 7, 12 and 18 days of incubation at 37°C. Colonies were defined as groups of 10 or more adherent cells.

Methylcellulose colony assay. FACS-isolated HSC/MPPs from three untreated chronic PV patients were plated in cytokine-supplemented methylcellulose medium (StemCell Technologies) as described previously (17) with or without the additional supplement of 50ug/mL CXCL4/PF4. Cells to be plated out with CXCL4/PF4 were pre-incubated with CXCL4/PF4 as described above. 29 cells were plated out per well, and three wells were evaluated for every PV patient and each condition tested. Colony growth was evaluated after 12 days of incubation at 37°C and 5% CO_2. Colony types were defined as previously described (18, 19).

Additional statistical analyses. Statistical significance of protein and RNA expression in different patient groups in HSC/MPPs and CMP/MEPs compared to controls was analyzed by 2way ANOVA (no missing values present) and mixed-effects analyses (missing values present), correcting for multiple comparisons using Dunnett's test. Student's t test was used for binary comparisons, applying two tails and paired tests in case of data in individual patient and matched control samples. Fisher's exact test was used for functional single cell CXCL4/PF4 assay data in individual patients. Regression analyses were

performed with ordinary least squares estimation, testing for significance of the estimated correlation by assuming an underlying F-distribution.

Antibody	Clone	Provider	Order number
Tricolor/phycoerythrin (PE)-Cy5-conjugated anti-hCD2	S5.5	ThermoFisherScientific-Invitrogen	CD0206
Tricolor/phycoerythrin (PE)-Cy5-conjugated anti-hCD3	7D6	ThermoFisherScientific-Invitrogen	MHCD03065
Tricolor/phycoerythrin (PE)-Cy5-conjugated anti-hCD4	S3.5	ThermoFisherScientific-Invitrogen	MHCD0406
Tricolor/phycoerythrin (PE)-Cy5-conjugated anti-hCD7	CD7-6B7	ThermoFisherScientific-Invitrogen	MHCD0706
Tricolor/phycoerythrin (PE)-Cy5-conjugated anti-hCD8	3B5	ThermoFisherScientific-Invitrogen	MHCD0806
Tricolor/phycoerythrin (PE)-Cy5-conjugated anti-hCD14	TuK4	ThermoFisherScientific-Invitrogen	MHCD1406
Tricolor/phycoerythrin (PE)-Cy5-conjugated anti-hCD19	SJ25-C1	ThermoFisherScientific-Invitrogen	MHCD1906
Tricolor/phycoerythrin (PE)-Cy5-conjugated anti-hCD56	MEM-188	ThermoFisherScientific-Invitrogen	MHCD5606
Phycoerythrin (PE)-Cy5 anti-hCD10	HI10a	BioLegend	312206
Phycoerythrin (PE)-Cy5 anti-hCD11b	ICRF44	BioLegend	301308
Phycoerythrin (PE)-Cy5 anti-hCD20	2H7	BioLegend	302308
Phycoerythrin (PE)-Cy5 anti-hCD235a	GA-R2	BD Biosciences	559944
PE-Cy7-conjugated anti-hCD34	8G12	BD Biosciences	348811
FITC-conjugated anti-hCD38	HIT2	BD Biosciences	555459
APC-conjugated anti-hCD123	6H6	ThermoFisherScientific-Invitrogen	17-1239-42
APC780-conjugated anti-hCD45RA	HI100	ThermoFisher Scientific-Invitrogen	47-0458-41
BV711-conjugated anti-hCD45RA	HI100	Biolegend	304138
PE-conjugated anti-hCXCL4/PF4	170138	R&D Systems	IC7952P
PE-conjugated monoclonal mouse IgG2B isotype control	133303	R&D Systems	IC0041P

REFERENCES

1. Amon S, et al. Sensitive Quantitative Proteomics of Human Hematopoietic Stem and Progenitor Cells by Data-independent Acquisition Mass Spectrometry. Mol Cell Proteomics. 2019;18(7):1454-67.

2. Perkins DN, Pappin DJ, Creasy DM, Cottrell JS. Probability-based protein identification by searching sequence databases using mass spectrometry data. Electrophoresis. 1999;20(18):3551-67.

3. Eng JK, Jahan TA, Hoopmann MR. Comet: an open-source MS/MS sequence database search tool. Proteomics. 2013;13(1):22-4.

4. Schubert OT, et al. Building high-quality assay libraries for targeted analysis of SWATH MS data. Nat Protoc. 2015;10(3):426-41.

5. Bruderer R, et al. Extending the limits of quantitative proteome profiling with data-independent acquisition and application to acetaminophen-treated three-dimensional liver microtissues. Mol Cell Proteomics. 2015;14(5):1400-10.

6. Choi M, et al. MSstats: an R package for statistical analysis of quantitative mass spectrometry-based proteomic experiments. Bioinformatics. 2014;30(17):2524-6.

7. Ritchie ME, et al. limma powers differential expression analyses for RNA-sequencing and microarray studies. Nucleic Acids Res. 2015;43(7):e47.

8. Picelli S, et al. Full-length RNA-seq from single cells using Smart-seq2. Nat Protoc. 2014;9(1):171-81.

9. Dobin A, et al. STAR: ultrafast universal RNA-seq aligner. Bioinformatics. 2013;29(1):15-21.

10. Liao Y, Smyth GK, Shi W. The Subread aligner: fast, accurate and scalable read mapping by seed-and-vote. Nucleic Acids Res. 2013;41(10):e108.

11. Love MI, Huber W, Anders S. Moderated estimation of fold change and dispersion for RNA-seq data with DESeq2. Genome Biol. 2014;15(12):550.

12. Cheung TH, Rando TA. Molecular regulation of stem cell quiescence. Nat Rev Mol Cell Biol. 2013;14(6):329-40.

13. Subramanian A, et al. Gene set enrichment analysis: a knowledge-based approach for interpreting genome-wide expression profiles. Proc Natl Acad Sci U S A. 2005;102(43):15545-50.

14. Garcia-Alonso L, Holland CH, Ibrahim MM, Turei D, Saez-Rodriguez J. Benchmark and integration of resources for the estimation of human transcription factor activities. Genome Res. 2019;29(8):1363-75.

15. Alvarez MJ, et al. Functional characterization of somatic mutations in cancer using network-based inference of protein activity. Nat Genet. 2016;48(8):838-47.

16. Knapp DJ, et al. Distinct signaling programs control human hematopoietic stem cell survival and proliferation. Blood. 2017;129(3):307-18.

17. Fritsch K, et al. Engineered humanized bone organs maintain human hematopoiesis in vivo. Exp Hematol. 2018;61:45-51 e5.

18. Manz MG, Miyamoto T, Akashi K, Weissman IL. Prospective isolation of human clonogenic common myeloid progenitors. Proc Natl Acad Sci U S A. 2002;99(18):11872-7.

19. Gregory CJ, Eaves AC. Human marrow cells capable of erythropoietic differentiation in vitro: definition of three erythroid colony responses. Blood. 1977;49(6):855-64.
